# The Twitter Social Mobility Index: Measuring Social Distancing Practices With Geolocated Tweets

**DOI:** 10.2196/21499

**Published:** 2020-12-03

**Authors:** Paiheng Xu, Mark Dredze, David A Broniatowski

**Affiliations:** 1 Malone Center for Engineering in Healthcare Center for Language and Speech Processing, Department of Computer Science Johns Hopkins University Baltimore, MD United States; 2 Department of Engineering Management and Systems Engineering The George Washington University Washington, DC United States

**Keywords:** COVID-19, social distancing, mobility, Twitter, social media, surveillance, tracking, travel, index

## Abstract

**Background:**

Social distancing is an important component of the response to the COVID-19 pandemic. Minimizing social interactions and travel reduces the rate at which the infection spreads and “flattens the curve” so that the medical system is better equipped to treat infected individuals. However, it remains unclear how the public will respond to these policies as the pandemic continues.

**Objective:**

The aim of this study is to present the Twitter Social Mobility Index, a measure of social distancing and travel derived from Twitter data. We used public geolocated Twitter data to measure how much users travel in a given week.

**Methods:**

We collected 469,669,925 tweets geotagged in the United States from January 1, 2019, to April 27, 2020. We analyzed the aggregated mobility variance of a total of 3,768,959 Twitter users at the city and state level from the start of the COVID-19 pandemic.

**Results:**

We found a large reduction (61.83%) in travel in the United States after the implementation of social distancing policies. However, the variance by state was high, ranging from 38.54% to 76.80%. The eight states that had not issued statewide social distancing orders as of the start of April ranked poorly in terms of travel reduction: Arkansas (45), Iowa (37), Nebraska (35), North Dakota (22), South Carolina (38), South Dakota (46), Oklahoma (50), Utah (14), and Wyoming (53). We are presenting our findings on the internet and will continue to update our analysis during the pandemic.

**Conclusions:**

We observed larger travel reductions in states that were early adopters of social distancing policies and smaller changes in states without such policies. The results were also consistent with those based on other mobility data to a certain extent. Therefore, geolocated tweets are an effective way to track social distancing practices using a public resource, and this tracking may be useful as part of ongoing pandemic response planning.

## Introduction

The outbreak of SARS-CoV-2, a coronavirus that causes the disease COVID-19, has caused a pandemic on a scale unseen in a generation. Without an available vaccine to reduce transmission of the virus, public health organizations and elected officials have called on the public to practice social distancing. Social distancing is a set of practices in which individuals maintain a physical distance to reduce the number of physical contacts they encounter [1,2]. These practices include maintaining a distance of at least six feet from other people and avoiding large gatherings [3]. At the time of this writing, in the United States, nearly every state had implemented statewide “stay-at-home” orders to enforce social distancing practices [4].

Social distancing is an important tool in the fight against COVID-19; however, its implementation by the general public can vary widely. Although a state governor may issue an order for the practice, individuals in different states may respond to this order in different ways. Courtemanche et al [5] showed that social distancing policies in the United States reduced the daily growth rate of COVID-19 cases. However, if we only consider the social distancing policy duration and daily confirmed cases, it is difficult to rule out potential confounders, including additional policies for wearing masks and improving hygiene as well as other social norms. Therefore, understanding actual reductions in travel and social contacts is critical to measuring the effectiveness of such policies. Using mobile phone data, Badr et al [6] found that mobility patterns were strongly correlated with decreased rates of COVID-19 case growth for the 25 most affected counties in the United States. These social distancing policies may remain in effect for an extended period of time. Thus, the public may begin to relax their practices, making additional policies necessary. Researchers showed the effectiveness of strict social distancing followed by testing and contact tracing by modeling mobility data from Cuebiq Inc in the Boston metropolitan area [7]. Additionally, epidemiologists have already modeled the impact of social distancing policies on the course of disease outbreaks [8-10]. These models may be more effective when incorporating actual measures of social distancing rather than assuming that official policies are implemented in practice.

It can be challenging to obtain data on the efficacy of social distancing practices, especially during an ongoing pandemic. In a recent Gallup poll that surveyed Americans, it was found that many adults are taking precautions to maintain distance from others [11]. However, while polling can provide insights, it cannot provide a solution. Polling is relatively expensive; thus, it is a poor choice for ongoing population surveillance practices and providing data on specific geographic locales (ie, US states and major cities) [12]. Additionally, polling around public health issues suffers from response bias, as individuals may overstate their compliance with established public health recommendations [13].

Over the past decade, analyses of social media and web data have been widely adopted to support public health objectives [14]. In this vein, several efforts have emerged over the past few months to track social distancing practices using these data sources. Google has released COVID-19 Community Mobility Reports [15] that use Google data to “chart movement trends over time by geography, across different categories of places such as retail and recreation, groceries and pharmacies, parks, transit stations, workplaces, and residential.” The Unacast Social Distancing Scoreboard uses data collected from 127 million monthly active users to measure the implementation of social distancing practices [16]. Researchers at the Institute for Disease Modeling have used data from Facebook’s Data for Good program to model the decline in mobility in the greater Seattle area and its effect on the spread of COVID-19 [17]. Using mobile phone data, the *New York Times* completed an analysis that showed that stay-at-home orders dramatically reduced travel; however, it was found that in states where such orders were not quickly enacted, residents continued to travel widely [18].

Identifying and isolating individuals who have potentially been exposed to a virus can blunt the spread of a pandemic. Contact tracing involves finding people who have had contact with an infected individual during the time the individual was contagious. In the current pandemic, several efforts have been made to develop digital contact tracing tools. Google and Apple announced a joint effort to build a Bluetooth-based contact tracing platform, which enhances the interoperability between Android and IOS devices using apps from public health authorities [19]. Singapore [20] and Australia [21] released similar apps that use Bluetooth to exchange “digital handshakes” to establish contacts. Many countries have developed their own contact tracing responses [22]. Li and Guo [23] presented a review of the development of contact-tracing apps for COVID-19. These efforts provide new and important opportunities to study social distancing and contact tracing in real time.

We present the Twitter Social Mobility Index, a measure of social distancing and travel patterns derived from public Twitter data. We used public geolocated Twitter data to measure how much a user travels in a given week. We computed a metric based on the standard deviation of a user’s geolocated tweets each week, and we aggregated these data over an entire population to produce a metric for the United States as a whole, for individual states, and for some US cities. We found that in the United States as a whole, there was a dramatic drop in travel in the later weeks of the study period, with travel between March 16 and April 27, 2020, showing the lowest amount since January 1, 2019, the start of our data set. Additionally, we found that travel reductions were not uniform across the United States but varied from state to state. However, there was no clear correlation between social mobility and confirmed COVID-19 cases at the state level. A key advantage of our approach is that unlike the other travel and social distancing analyses referenced above, we rely on entirely public data, which enables others to replicate our findings and explore different aspects of these data. Additionally, because Twitter contains user-generated content in addition to location information, future analyses can correlate users’ attitudes, beliefs, and behaviors with changes in social mobility.

One concern regarding the mining of social media data is user privacy. Unlike the data used by the companies described above, all the data we used is publicly available. Users choose to post their location data to Twitter publicly; therefore, these data are accessible to all users. However, while the location data are public, the potential remains for violating user privacy and producing unintended consequences for users, such as highlighting users who are failing to social distance. To ensure privacy in our index, we aggregated all mobility metrics to produce population-level analyses. None of our work considers the identity of individual users, and we removed identifiable user information from the distributed data aggregations. Furthermore, we caution others who pursue work similar to ours to consider privacy ramifications for users when collecting new data and conducting similar analyses.

There is widespread recognition that real-time tweets from millions of users can yield insights into a variety of population-level trends. Our study follows a tradition of using this insight to develop population-level indices and measures from Twitter data. Previous work includes tracking population-level sentiment as an economic indicator that can track stock price [24], political indices that reflect the popular opinion on major socioeconomic issues [25] or opinions about political candidates [26,27], and measures of pop culture such as reception of entertainment programs [28]. The Twitter Social Mobility Index is a measure of this kind, aggregating Twitter data from millions of people to produce real-time measurements of social distancing.

There is a long line of work on geolocation prediction for Twitter, which requires inferring a location for a specific tweet or user [29-32]. This includes work on patterns and trends in geotagged Twitter data [33]. Although most of these works focus on inferences of users’ current locations and thus are not suitable for tracking user movements, there may be opportunities to combine these methods with our approach.

Many studies have analyzed Twitter geolocation data to study population movements. Hawelka et al [34] demonstrated a method for computing global travel patterns from Twitter, and Dredze et al [35] adapted this method to support efforts in combating the Zika virus epidemic. Several studies have used human mobility patterns from Twitter data [36-39]. These studies include analyses of urban mobility patterns [40-42]. Finally, some of these analyses considered mobility patterns around mass events [43].

Our findings are presented on a website [44], and we will continue to update our analysis during the COVID-19 pandemic.

## Methods

### Data Source

Twitter offers several ways in which a user can indicate their location. If a user is tweeting from a GPS-enabled device, they can attach their exact coordinates to that tweet. Twitter can then display the specific place that corresponds to these coordinates to the user and also provide it in their application programming interface (API). Alternatively, a user can explicitly select a location, which can be a point of interest (eg, a coffee shop), neighborhood, city, state, or country. If the tweet is public, this geolocation information is supplied with the tweet.

We used the Twitter streaming API [45] to download tweets based on location. We used a bounding box that covered the entire United States, including US territories. We used data from this collection starting on January 1, 2019, and ending on April 27, 2020. In total, the data set included 3,768,959 Twitter users and 469,669,925 tweets posted in the United States.

### Location Data

We processed the two types of geolocation information described in the previous section.

#### Coordinates

We processed the exact coordinates (latitude and longitude) provided by the user (the “coordinates” field in the Twitter JavaScript Object Notation [JSON] object). Approximately 8% of our data included coordinates.

#### Place

The “place” field in the Twitter JSON object indicates a known location in which the tweet was authored. A place can be a point of interest (eg, a specific hotel), a neighborhood (eg, downtown Jacksonville), a city (eg, Kokomo, IN), a state (eg, Arizona), or a country (eg, the United States). The place object contains a unique ID, a bounding box, a country, and a name. More information about the location is available from the Twitter Geo API. A place is provided with a tweet in either of two conditions. First, Twitter can identify the coordinates provided by the user as occurring in a known place. Second, the user can manually select a place when authoring the tweet.

Because coordinates give a more precise location, we used them instead of place when available. If only a place was available, we assumed that the user was in the center of the place, as given by the place’s bounding box.

For points of interest and neighborhoods, Twitter only provides the country in the associated metadata. Although in some cases, the city can be parsed from the name and the state inferred, we opted to exclude these places from our analysis for states. The full location details can be obtained from querying the Twitter API; however, due to the magnitude of the data in our analysis, this task would have been too time-consuming. This limitation excluded approximately 1.8% of our data.

We performed analyses for the 50 most populous US cities. For these analyses, we included points of interest that c the city name in their names, such as “New York City Center.” Specifically for New York City, we included places that corresponded to each of the five New York City boroughs (Brooklyn, Manhattan, Queens, Staten Island, and the Bronx).

In summary, for each geolocated tweet, we obtained an associated latitude and longitude.

### Computing Mobility

We defined the Twitter Social Mobility Index as follows. For each user, we collected all locations (coordinates) in a 1-week period, where a week starts on Monday and ends the following Sunday. We denoted the coordinate sequence as 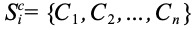, where *C_j_* is the coordinate at time *j* in week *i* and *n* is the number of coordinates in that week. We computed the centroid of all of the coordinates and considered this the “home” location for the user. We then measured the distance between each location and the centroid for that week. To determine distance, we measured the geodesic distance in kilometers between two adjacent records, *C_j_* and *C_j+1_*, using geopy [46], resulting in a distance sequence of 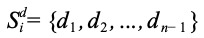. After collecting the distances, we measured the standard deviations of these distances. Formally, we defined Twitter Social Mobility Index *M* for each user as

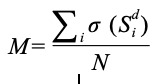



where *σ*(·) is the standard deviation operator and *N* is the number of weeks considered for the measure. We measured mobility in kilometers.

In summary, this measure reflects the area and regularity of travel for a user rather than the raw distance traveled. Therefore, a user who takes a long trip with a small number of check-ins would have a larger social mobility measure than a user with many check-ins who traveled in a small area. Because the measure is sensitive to the number of check-ins, it reflects when people have fewer check-ins during the pandemic.

We aggregated the results by week by taking the mean measure of all users in a given geographic area. We also present results for a 7-day moving average aggregation as a measure of daily movement. We recorded the variance of these measures to study the travel variance in the population, which indicates if travel is reduced overall but not for some users.

We produced aggregate scores by geographic area for the United States as a whole, for each US state and territory, and for the 50 most populous cities in the United States. We determined the geographic area of a user based on their centroid location for all times in our collection.

We computed the social mobility index for each day and week between January 1, 2019, and April 27, 2020. We selected the date of March 16, 2020, as the start of social distancing on the national level, although individual states implemented practices at different times. Therefore, we divided the data into two time periods: before social distancing (January 1, 2019, to March 15, 2020) and after social distancing (March 16, 2020, to April 27, 2020).

We then computed the group level reduction in social mobility by considering the average values as follows:

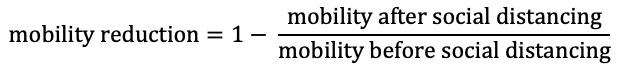



We also computed the reduction for each user and then tracked the median value, number of users active in both periods, and proportion of active users who completely reduced their mobility. We conducted a similar analysis for seasonal effects by comparing mobility after social distancing with mobility during the same period in 2019.

To address sparse data issues in our data set, we excluded users with fewer than 3 geolocated tweets overall and excluded the weekly record for a user if they had fewer than 3 geolocated tweets in that week. Additionally, due to data loss in our data collection process, we removed two weeks that contained far less data than the other time periods by taking a 99.75% confidence limit on the number of users and records.

## Results

### Social Mobility Index

Table 1 shows the Twitter Social Mobility Index measured in kilometers for every state and territory in the United States and the United States as a whole. City results are shown in Table 2. We also included the rank of location by the group level reduction.

**Table 1:** Reductions of mobility for all US states and territories and for the United States. Ranks are based on group level reduction.

Location	Mobility (kilometers)		Group level reduction (%)	User-level reduction (%)		Rank
	Before distancing	After distancing		Median reduction	Median seasonal reduction	
AK	109.76	25.47	76.80	99.84	63.73	1
AL	48.04	22.57	53.03	84.47	72.94	47
AR	50.54	23.15	54.19	91.87	76.81	45
AZ	62.85	23.47	62.66	93.69	85.55	26
CA	78.58	29.60	62.33	96.65	91.35	29
CO	72.23	24.47	66.12	98.2	93.37	12
CT	45.51	14.89	67.28	96.29	89.25	8
DC	77.67	19.74	74.58	100.00	97.75	2
DE	43.63	13.61	68.81	93.44	85.08	7
FL	76.99	32.24	58.13	92.38	82.92	42
GA	65.64	27.11	58.70	85.26	78.00	39
HI	147.61	70.75	52.07	97.69	89.21	51
IA	50.42	20.59	59.17	95.91	89.82	37
ID	70.77	33.36	52.86	94.12	78.19	49
IL	55.59	19.38	65.15	98.71	93.01	16
IN	45.86	17.15	62.60	97.19	89.61	27
KS	65.50	23.19	64.60	97.03	81.57	19
KY	44.67	15.31	65.74	93.93	83.42	13
LA	45.98	19.39	57.83	86.13	77.76	43
MA	58.69	17.64	69.95	98.83	93.93	5
MD	46.10	15.19	67.04	94.80	88.67	9
ME	59.68	22.45	62.38	93.77	78.53	28
MI	56.24	20.96	62.72	96.84	90.42	25
MN	64.01	21.68	66.13	98.36	91.34	11
MO	52.27	20.08	61.59	95.89	88.65	31
MS	50.24	24.36	51.51	79.09	69.11	52
MT	69.93	32.96	52.86	90.17	65.58	48
NC	52.11	19.73	62.14	94.27	85.26	30
ND	65.77	23.65	64.04	99.71	97.21	22
NE	55.11	21.88	60.29	99.95	91.40	35
NH	55.09	19.48	64.64	96.26	85.35	18
NJ	49.27	14.62	70.33	97.28	93.41	4
NM	58.20	24.23	58.37	95.66	73.14	41
NV	80.25	33.19	58.64	93.42	85.00	40
NY	71.17	24.57	65.48	98.94	94.20	15
OH	44.88	15.73	64.95	94.81	88.68	17
OK	52.34	24.69	52.83	88.38	76.99	50
OR	71.12	25.97	63.49	97.51	92.68	24
PA	54.40	19.45	64.24	97.59	89.85	20
PR	44.96	14.94	66.77	97.26	90.38	10
RI	46.80	14.50	69.01	96.74	90.55	6
SC	48.28	19.85	58.88	86.03	77.92	38
SD	68.41	31.52	53.92	95.91	86.66	46
TN	56.77	21.83	61.55	94.89	85.89	32
TX	73.24	28.60	60.95	93.81	84.18	34
UT	68.43	23.62	65.49	93.56	91.50	14
VA	57.37	22.33	61.07	95.62	87.51	33
VI	132.16	47.57	64.00	98.66	87.72	23
VT	56.84	20.33	64.23	96.35	86.70	21
WA	75.34	21.31	71.71	98.43	95.72	3
WI	56.32	22.68	59.74	96.88	91.75	36
WV	46.59	20.02	57.02	88.95	82.40	44
WY	71.64	44.03	38.54	84.95	50.90	53
United States	65.59	25.04	61.83	95.86	88.36	N/A^a^

^a^N/A: not applicable.

**Table 2:** Reduction of mobility for top 50 United States cities by population. Ranks are based on group level reduction.

Location	Mobility (kilometers)		Group level reduction (%)	User level reduction (%)		Rank
	Before distancing	After distancing		Median reduction	Median seasonal reduction	
New York City	86.37	29.91	65.38	99.70	96.69	27
Los Angeles	103.16	40.86	60.39	98.69	93.87	40
Chicago	64.09	19.87	69.00	99.96	94.58	14
Houston	53.70	21.50	59.96	97.04	88.00	41
Phoenix	60.07	19.12	68.17	96.32	91.08	18
Philadelphia	54.80	17.70	67.71	99.16	93.70	19
San Antonio	45.43	15.93	64.93	99.00	91.33	28
San Diego	79.21	28.19	64.41	98.67	92.77	30
Dallas	63.92	21.85	65.81	95.48	89.32	25
San Jose	60.63	14.82	75.55	99.88	97.34	2
Austin	72.50	22.84	68.50	99.66	94.66	17
Jacksonville	47.06	26.87	42.90	96.60	92.92	50
Fort Worth	51.67	19.68	61.92	95.33	85.72	37
Columbus	44.67	14.73	67.02	96.91	93.15	22
San Francisco	113.77	31.99	71.89	99.93	98.94	8
Charlotte	58.13	20.90	64.04	96.26	89.83	31
Indianapolis	46.50	14.53	68.76	99.26	91.85	15
Seattle	98.92	21.64	78.12	99.98	99.06	1
Denver	81.11	23.08	71.55	99.05	96.30	9
Washington	80.26	22.12	72.43	99.93	97.27	7
Boston	77.58	27.47	64.59	99.42	96.40	29
El Paso	51.10	21.50	57.92	100.00	95.97	44
Detroit	53.94	22.38	58.50	94.89	83.68	43
Nashville	72.83	23.94	67.13	98.45	94.88	21
Portland	78.91	24.81	68.56	99.45	96.81	16
Memphis	48.64	18.41	62.15	98.65	86.75	35
Oklahoma City	46.07	16.78	63.57	91.34	75.19	33
Las Vegas	80.21	35.69	55.50	94.87	83.90	47
Louisville	45.52	12.97	71.51	94.31	77.68	10
Baltimore	45.61	11.66	74.43	96.10	89.37	4
Milwaukee	52.01	22.78	56.19	97.01	91.86	46
Albuquerque	51.04	16.88	66.93	98.95	75.81	23
Tucson	53.58	23.10	56.89	95.73	84.48	45
Fresno	37.39	10.84	71.02	96.06	89.20	11
Mesa	48.77	21.72	55.47	92.40	71.33	48
Sacramento	62.14	25.45	59.05	94.82	94.47	42
Atlanta	87.90	33.39	62.02	93.50	86.36	36
Kansas City	62.93	17.23	72.61	98.30	96.54	6
Colorado Springs	64.82	23.55	63.67	99.47	95.66	32
Miami	114.33	55.77	51.22	97.55	88.56	49
Raleigh	51.62	15.24	70.47	97.79	89.51	12
Omaha	49.99	15.38	69.24	100.00	93.72	13
Long Beach	54.97	20.51	62.70	93.33	89.75	34
Virginia Beach	48.91	18.92	61.33	96.35	88.38	39
Oakland	87.36	22.26	74.52	98.41	96.26	3
Minneapolis	69.67	18.72	73.14	99.14	94.21	5
Tulsa	48.54	18.51	61.85	99.89	93.20	38
Arlington	56.42	18.27	67.62	97.58	93.25	20
Tampa	70.50	23.55	66.59	94.48	83.23	24
New Orleans	55.96	19.18	65.73	97.00	88.75	26

**Figure 1:**
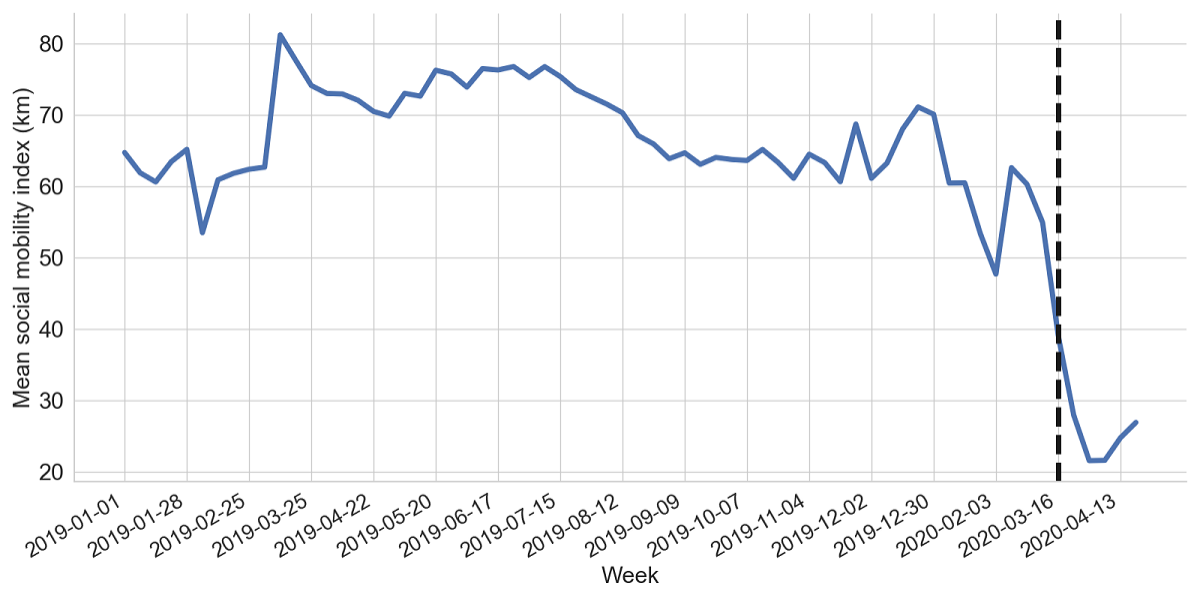
Mean social mobility index (kilometers) in United States from January 1, 2019, to April 27, 2020. Weeks with missing data are excluded from the figure.

We observed that the overall drop in mobility across the United States was large (61.83%). Figure 1 shows the weekly social mobility index for the United States for the entire time period of our data set. The figure reflects a massive drop in mobility starting in March, and the four most recent weeks showed the lowest mobility on record in our data set. Second, every US state and territory saw a drop in mobility, ranging from 38.54% to 76.80% of travel compared to the numbers before March 16, 2020. However, the variance by state was high. States that were early adopters of social distancing practices ranked highly on the reduction in travel, such as Washington (3) and Maryland (9). In contrast, the eight states that had not implemented statewide orders as of the start of April [4] ranked poorly, namely Arkansas (45), Iowa (37), Nebraska (35), North Dakota (22), South Carolina (38), South Dakota (46), Oklahoma (50), Utah (14), and Wyoming (53). We observed similar trends in the city analysis; however, the median users in cities had a larger mobility reduction than the users in states.

In addition to the group-level mobility travel reduction, we examined the distribution of user-level travel reduction. For this analysis, we only considered the subgroup of users who had at least two check-ins in both periods. The median values for the reduction distribution were close to 100% for most states. The median values for seasonal reduction were all smaller but still suggested that people substantially reduced their mobility during the pandemic. Moreover, in the United States, 40% of the 818,213 active users completely reduced their mobility (ie, the mobility reduction was 100%). In contrast, during the same period in 2019, a 31% reduction was seen among 286,217 active users.

The White House announced “Slow the Spread” guidelines for persons to take action to reduce the spread of COVID-19 on March 16, 2020 [47]. Of the states, 49.06% (26/53) had their largest mobility drop in the week of March 16-22, 2020, and 22.64% (12/53) had their largest drop in the following week. We computed a moving average of daily mobility data and used an offline change point detection method [48] on this trend. In 2020, 62.26% of the change points occurred after the national announcement date but before the dates on which individual state policies were enacted. This suggests that the national announcement had a larger effect compared to state policies, which is a similar finding to that of a mobile phone–based mobility analysis of four large cities [49]. We also observed that among the 40 states that announced stay-at-home policies, 92.5% (37) of the states had a more stationary daily mobility time series before the policy announcement date compared to the mobility time series over the entire time period, suggesting a rapid mobility change during the pandemic.

**Figure 2:**
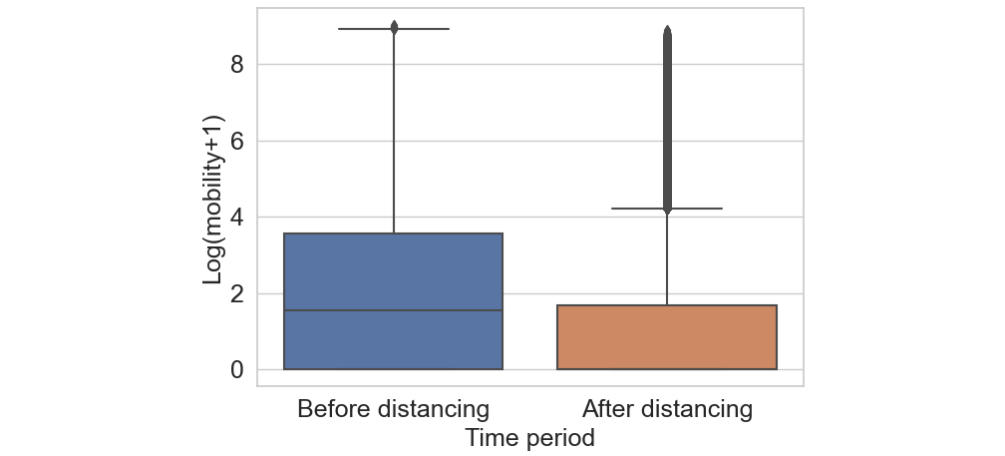
Box plots showing the user distributions of the mean social mobility index (kilometers) before and after social distancing measure were enacted in the United States.

Finally, Figure 2 shows a box plot of the mobility variance across all users in a given time period. The distribution is long-tailed with numerous zeros; therefore, we took the log of 1 plus each mobility index. Although mobility was reduced in general, some users still showed a lot of movement, which suggests that social distancing is not being uniformly practiced. These results clearly demonstrate that our metric can track drops in travel, suggesting that it can be used as part of ongoing pandemic response planning.

### Correlations

To investigate the factors that explain our Twitter Social Mobility Index and how well the index tracks COVID-19 cases compared to other relevant factors, we performed a correlation analysis on our data. We computed the daily infection rate by dividing the number of new confirmed COVID-19 cases in each US state [50] by the population of the state. We compared the daily infection rate with the social mobility index and the trends in the state characteristics category from [51]. We first ran a correlation analysis for the following trends: state size in square miles, population density per square mile, unemployment rate (2018), percentage of the population living under the federal poverty line (2018), number of homeless individuals (2019), percentage of the population at risk for serious illness due to COVID-19, and number of all-cause deaths (2016). We selected these measures to track the size of the state, economic activity, and composition of the population, which were studied in a similar correlation analysis of other countries [52]. These measures may change how far people typically travel in a given state.

**Figure 3:**
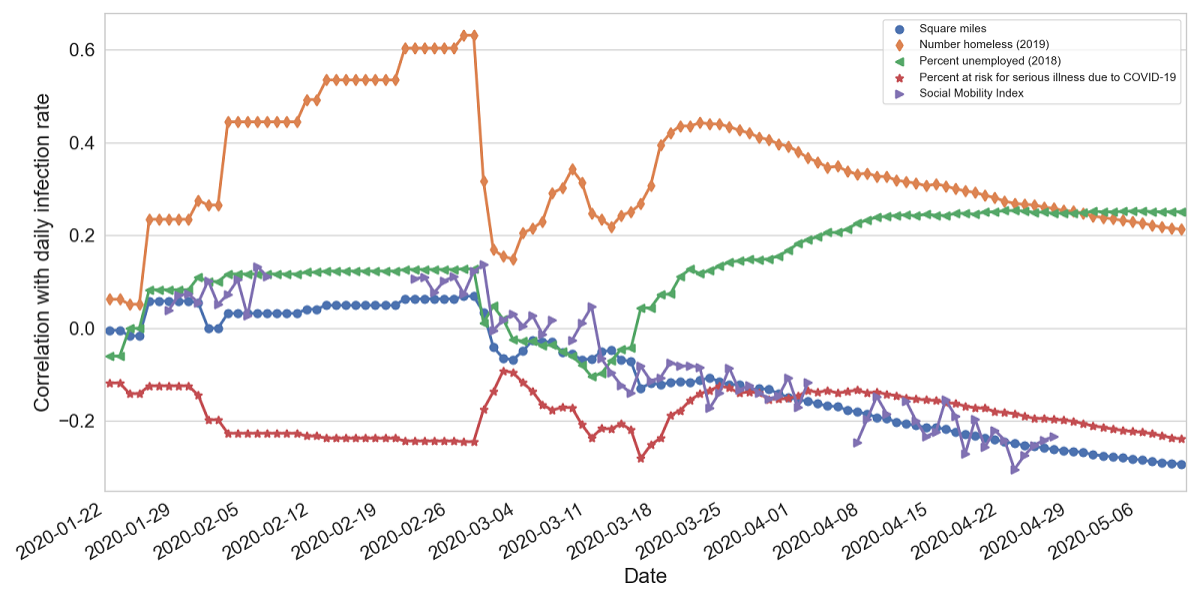
Pearson correlations between daily COVID-19 infection rates and various factors at the state level.

**Figure 4:**
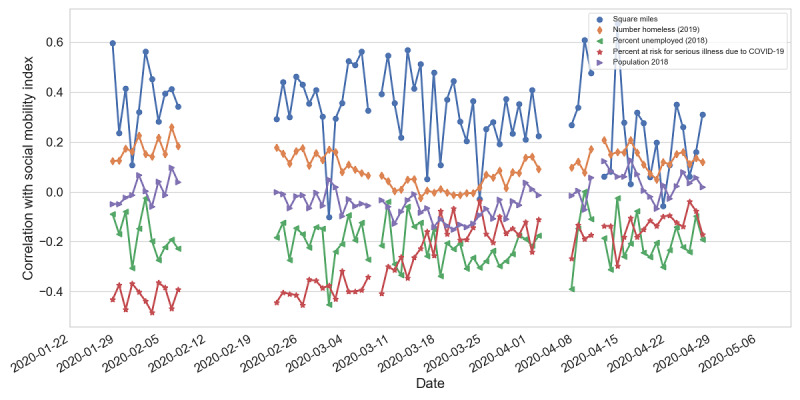
Pearson correlations between the social mobility index and various factors at the state level.

In Figure 3 and Figure 4, we show the characteristics that have high correlation with either the number of confirmed cases or the mobility index. These characteristics were the size of the state in square miles, the number of homeless individuals (2019), the unemployment rate (2018), and the percentage of the population at risk for serious illness due to COVID-19.

For each day, we computed the correlations between the daily infection rate and the above data by state.

Figure 3 shows the correlations by day. We adopted the infection rate because the raw number of confirmed cases is not as informative, as the population has the highest correlation. However, the most significant factors in the early stage were still population-related factors (eg, the number of homeless people). We did not see significant correlations with other factors, including the social mobility index. Starting from mid-March, we observed trends of increasing correlation with the unemployment rate, size of the state, and social mobility index; however, these correlations were not significant (absolute correlation values <.5). A fluctuation occurred in the middle of the period, when states started to report confirmed cases of COVID-19.

We conducted a similar correlation analysis between each data source and the social mobility index, as shown in Figure 4. As expected, geographical state size showed the highest positive correlation. We also observed that the number of people at risk for serious illness due to COVID-19 had a negative correlation at the early stage of the pandemic.

Table 3 demonstrates the effects of various restriction policies on confirmed cases by running a similar correlation analysis on the cumulative confirmed cases for each state on May 10, 2020. The policy types follow the data from [51]. We used the time difference (in days) between May 10, 2020, and the policy release date as the input for the analysis, and we assigned a negative value (–1000) to states that had not announced a policy. The factor with the highest correlation with the social mobility index is the declaration of a state of emergency, which is the broadest type of policy.

**Table 3:** Pearson correlations between the cumulative number of confirmed COVID-19 cases on May 10, 2020, and the dates on which policies were released in each state.

Policy	Correlation	*P* value
State of emergency	0.2587	.07
Date banned visitors to nursing homes	0.151	.29
Stay-at-home or shelter-in-place order	0.1507	.29
Evictions frozen	0.1411	.32
Nonessential businesses closed	0.1359	.34
Gyms closed	0.0765	.59
Movie theaters closed	0.0737	.61
Day cares closed	0.0563	.70
Restaurants closed except takeout	0.0341	.81
Kindergarten to 12th grade schools closed	–0.0821	.57

## Discussion

We present the Twitter Social Mobility Index, a measure of social mobility based on public geolocated tweets. Our analysis shows that there was a large drop in mobility overall in the United States. However, the drop was inconsistent and varied significantly by state. It appears that states that were early adopters of social distancing practices experienced more significant drops than states that had not yet implemented these practices.

Several limitations of using geo-tagged tweets as the subject of our study must be kept in mind. First, users on Twitter and other social media platforms are not representative of the general population. Their demographics, such as age, race, ethnicity, education level, income, and political affiliation, do not perfectly mirror the larger population. In the United States, Twitter users are younger, more educated, have higher incomes, and are more likely to identify as Democrats than the general public [53,54]. Therefore, while our sample of users is large, it is highly biased.

Second, not all users are equally likely to use geotagging features on Twitter, and they may use the features in different ways. For example, in a previous study [32], demographic differences were found in the groups of people who used the two different types of geolocation information (ie, coordinates and place). GPS-tagged tweets are posted more often by young people and by women compared to tweets with self-reported locations.

Third, while we obtained access to millions of geotagged tweets, this is still a relatively small proportion of the total number of nongeotagged tweets on the platform, and it is also small compared to private measures of social mobility computed by companies such as Google and Apple.

Fourth, a small proportion of geotagged tweets report fake geolocation information. However, we believe that this is a negligible problem, as previous work found the rate of fake geolocation to be around 0.22% on social media in general [55] and even lower on Twitter. In our preliminary analysis, we considered mobility data based on GPS from mobile devices alone while excluding place information, as this method has greater precision. However, our results with these limited data were similar to our results with the full data set, except that they were less stable. Therefore, we decided to include all location data.

Despite these limitations, our results produced metrics that align with expected trends given national social distancing guidelines and related statewide policies. This suggests that there is sufficient information in our data to overcome these limitations. Additionally, the public nature of Twitter data has advantages over proprietary and private data sources. More work is needed to compare our mobility trends with those of other data sources.

Our work on this data is ongoing, and there are several directions that warrant further study. First, as states begin to reopen and some states maintain restrictions, tracking changes in population behaviors will be helpful in making policy decisions. Second, we focused on the United States; however, Twitter data provides sufficient coverage to replicate our analysis for many countries. Third, tweet content exists for each user in the data set; this content can reflect the user's attitudes, beliefs, and behaviors. Studying these factors together with users’ mobility reduction could yield further insights. Our findings are presented on a website [44], and we will continue to update our analysis during the pandemic.

## Abbreviations

API: application programming interface
JSON: JavaScript Object Notation
